# Cross-cultural adjustment to the United States: the role of contextualized extraversion change

**DOI:** 10.3389/fpsyg.2015.01650

**Published:** 2015-10-29

**Authors:** Mengqiao Liu, Jason L. Huang

**Affiliations:** ^1^Department of Psychology, Wayne State University, DetroitMI, USA; ^2^School of Human Resources and Labor Relations, Michigan State University, East LansingMI, USA

**Keywords:** extraversion, personality change, contextualized personality, cross-cultural adjustment, international students, United States, cross-cultural motivation, latent growth model

## Abstract

Personality traits can predict how well-sojourners and expatriates adjust to new cultures, but the adjustment process remains largely unexamined. Based on recent findings that reveal personality traits predict as well as respond to life events and experiences, this research focuses on within-person change in contextualized extraversion and its predictive validity for cross-cultural adjustment in international students who newly arrived in US colleges. We proposed that the initial level as well as the rate of change in school extraversion (i.e., contextualized extraversion that reflects behavioral tendency in school settings) will predict cross-cultural adjustment, withdrawal cognitions, and school satisfaction. Latent growth modeling of three-wave longitudinal surveys of 215 new international students (54% female, *M*_age_ = 24 years) revealed that the initial level of school extraversion significantly predicted cross-cultural adjustment, (lower) withdrawal cognitions, and satisfaction, while the rate of change (increase) in school extraversion predicted cross-cultural adjustment and (lower) withdrawal cognitions. We further modeled global extraversion and cross-cultural motivation as antecedents and explored within-person change in school extraversion as a proximal factor that affects adjustment outcomes. The findings highlight the malleability of contextualized personality, and more importantly, the importance of understanding within-person change in contextualized personality in a cross-cultural adjustment context. The study points to more research that explicate the process of personality change in other contexts.

## Introduction

In today’s global economy, adapting and adjusting to new cultures as sojourners and expatriates have become increasingly important. International corporations frequently send individuals to work in foreign countries for an extended period of time, and the success of such foreign assignment is anything but guaranteed (Brookfield Global Relocation Services, 2012). Among the factors that lead to expatriate outcomes, cross-cultural adjustment is a crucial contributor to expatriate success, while inability to adjust is linked to expatriate’s early return and inadequate performance (Naumann, 1992; Shaffer et al., 1999; Anderson, 2005).

Given the importance of understanding the antecedents of cross-cultural adjustment, there has been a growing interest in identifying individual characteristics to predict cross-cultural adjustment (Arthur and Bennett, 1995; Ones and Viswesvaran, 1999). In particular, personality (e.g., Swagler and Jome, 2005; Sri Ramalu et al., 2010) and cross-cultural motivation (e.g., Templer et al., 2006) have been shown as antecedents of successful adjustment. However, following research on personality change (e.g., Vaidya et al., 2002; Lüdtke et al., 2011), it is likely that sojourners experience personality change in response to the changes they encounter in a new cultural environment. More importantly, it remains to be seen whether such change can predict cross-cultural adjustment.

The goal of the current research is to study contextualized extraversion change in the cross-cultural adjustment process. We situate our investigation in a particular population: newly arrived international students in US colleges and universities, given the unique dual challenge faced by this population. On the one hand, international students, like all sojourners, undergo the process of adjusting to a foreign culture. On the other hand, international students need to manage the academic (e.g., Credé and Niehorster, 2012) and social demands (Ross et al., 1999) that can have a substantial impact on their long-term career outcomes.

This study makes several theoretical contributions to the personality and work adjustment literature, accompanied by practical implications for organizations. First, by recognizing the malleable aspect of personality, organizational researchers can better understand how personality changes and the impact of these changes on vocational and career adjustment. Second, examining personality changes in the cross-cultural context will lay the ground work for illuminating personality changes in other important, specific contexts pertaining to work-related transitions and adaptation.

### Cross-cultural Adjustment

In the past few decades, there has been a growing interest in studying personality to predict cross-cultural adjustment. For instance, Swagler and Jome (2005) showed that high levels of agreeableness and conscientiousness, as well as low levels of neuroticism, were linked to better psychological adjustment among American Sojourners in Taiwan. In addition, sociocultural adjustment was predicted by high levels of extraversion. Similar results were found by Sri Ramalu et al. (2010), where high levels of agreeableness and extraversion were associated with better general cross-cultural adjustment among a diverse sample of expatriates in Malaysia, while greater conscientiousness and openness to experience were linked to better work adjustment. In addition, Peltokorpi (2008) revealed that emotional stability was related to better adjustment in both non-work related (interaction and general living) and work related adjustment among expatriates from 21 countries in Japan.

**FIGURE 1:**
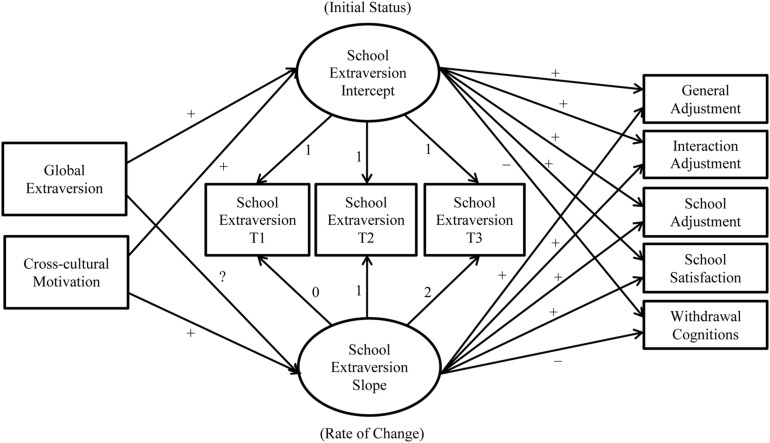
**Model 1 with hypotheses**.

Although researchers have examined various personality traits that predict cross-cultural adjustment, we focus on extraversion and cross-cultural motivation in our current research model (see **Figure 1**) as distal antecedents. We briefly review the influence of extraversion and cross-cultural motivation on adjustment outcomes below.

In the current study, we focus on the personality domain of extraversion as a lens to understanding the process of cross-cultural adjustment. Extraversion describes the extent to which a person is assertive, warm, excitement-seeking, and people-oriented (Costa and McCrae, 1992). Research has shown that extraverts tend to seek out more social activities (e.g., Argyle and Lu, 1990; Asendorpf and Wilpers, 1998), grow a bigger social network (e.g., Asendorpf and Wilpers, 1998), and create a more positive social environment (e.g., Eaton and Funder, 2003). Not only are extraverts more sociable, they also enjoy social activities to a greater extent. Studies have shown that extraverted individuals tend to report higher intimacy, higher satisfaction, and less conflict in social interactions (e.g., Barrett and Pietromonaco, 1997; White et al., 2004). Given its relevance in social interactions, extraversion in a cross-cultural setting may drive sojourners to seek out more interpersonal relationships, receive more social support, and gain satisfaction via interacting with others. In addition, extraverts also tend to be happier than introverts, owing to the positive relationship between extraversion and positive affect (Costa and McCrae, 1992; Lucas and Baird, 2004). In line with this reasoning, extraversion has been shown to significantly predict sojourner (Swagler and Jome, 2005) and expatriate (Sri Ramalu et al., 2010) adjustment and satisfaction.

Yet another reason to highlight the role of extraversion in cross-cultural adjustment lies in America’s overall “extraverted culture.” Data has suggested that America has one of the highest average scores on extraversion in the world (McCrae and Terracciano, 2005). According to the person-environment fit literature that emphasizes the congruence between individual characteristics and the environment (Kristof-Brown and Guay, 2011), extraversion should be especially relevant and predictive of adjustment in the US.

Cross-cultural adjustment can be challenging. Thus, successful cross-cultural adjustment requires motivation that drives one’s continuous effort in learning and engaging in the new environment. Cross-cultural motivation is defined as “the capability to direct attention and energy toward learning about and functioning in situations characterized by cultural differences” (Ang et al., 2007). It is one of the three dimensions in the Cultural Intelligence framework that captures two related aspects, namely cross-cultural self-efficacy and cross-cultural intrinsic motivation (Ang et al., 2007; Chen et al., 2010). First, individuals with high cross-cultural motivation tend to be more self-efficacious in their adaptive capability. According to the theory of self-efficacy, individuals who believe in their capability tend direct more attention and effort in gathering information and developing strategies to meet the challenges (Bandura, 2002). Thus, in a cross-cultural setting, high motivation can enable sojourners to better channel their effort and knowledge into understanding the local culture and behave accordingly. Second, cross-cultural motivation reflects higher intrinsic interests in being part of the cross-cultural experiences. Compared to those with little or no motivation, highly motivated individuals enjoy social interactions with people from other cultures, and are more likely to adjust behaviors to achieve smooth and successful encounters (Chen et al., 2010). Research evidence suggests that cross-cultural motivation can enable better adjustment and adaptation in a foreign culture (Templer et al., 2006; Ang et al., 2007; Chen et al., 2010). Therefore, we posit that individuals with higher levels of cross-cultural motivation will also have better cross-cultural adjustment outcomes.

### Personality Change

Cross-cultural adjustment is a process where a person interacts with and adapts to a foreign environment. Although the existing literature has shed light on the impact of personality on cross-cultural adjustment, the focus on traits as static dispositions ignores the potential *process* by which one experiences changes in personality during cross-cultural adaptation. Given the malleable aspect of personality that has been demonstrated in recent research (Caspi and Roberts, 2001), we argue that change in personality can be used as an appropriate lens to investigate the process of cross-cultural adjustment.

The person-environment interactional approach of personality development recognizes the active role people take in their environment, and emphasizes the interactive dynamics between the traits and environmental contexts in shaping personality changes (Fraley and Roberts, 2005). On the one hand, *selection effects* posit that personality traits predict life events, such that people with different personality traits would select themselves into different events or be selected by others into different situations (Headey and Wearing, 1989; Roberts and Wood, 2006). On the other hand, *socialization effects* refer to the influence of life events on personality traits, such that personality changes are reactions to these events (Roberts and Mroczek, 2008; Roberts et al., 2008). Longitudinal studies have supported both effects (e.g., Vaidya et al., 2002; Specht et al., 2011; Boyce et al., 2015). For instance, in a two-wave longitudinal study, Vaidya et al. (2002) demonstrated support for selection effects, such that college students who scored higher on initial levels of extraversion, agreeableness, and conscientiousness were more likely to experience positive events later on, whereas negative events were predicted by lower initial levels of agreeableness and conscientiousness, as well as higher initial levels of neuroticism. Socialization effects were also supported, linking positive events (Time 1) with increases in extraversion and negative events (Time 1) with increases in neuroticism over time. Similar patterns of results were shown in a 4-year longitudinal study (Lüdtke et al., 2011). Comparing samples of young adults who followed different career paths, initial levels of personality traits had a significant impact on career choices (i.e., attending college or vocational training), while experiences and events in different careers also predicted changes in personality traits among these individuals. Socialization effects were also evident when linking work and social experiences to personality changes. For instance, individuals with higher work participation or advances in status also reported increases in domains of conscientiousness (agency and norm adherence; Roberts, 1997; Roberts et al., 2001) and in the social dominance facet of extraversion (self-confidence and assertiveness; Clausen and Gilens, 1990). In a sample of German adults, Boyce et al. (2015) also showed support for the socialization effects in the context of unemployment, such that individuals who had undergone unemployment experienced significant patterns of change in agreeableness, conscientiousness, and openness to experience. Experiences from social relationships can also contribute to change in personality traits, such that first time in a romantic relationship was associated with increases in extraversion and conscientiousness, as well as decreases in neuroticism (e.g., Neyer and Lehnart, 2007; Lehnart et al., 2010; however, see Asendorpf and Wilpers, 1998). Therefore, it can be concluded that personality traits predict as well as respond to life events and experiences.

Building upon the empirical findings on personality and its predictive value, researchers have started to use personality change to aid the prediction of various outcomes. For instance, Mroczek and Spiro (2007) found that mortality among aging men was predicted by both high initial levels and increases in neuroticism (*N* = 1,663). In addition, Siegler et al. (2003) showed that gains in hostility from college to midlife were linked to a wide range of negative outcomes, such as social isolation, obesity, as well as negative changes in economic and work life.

In sum, the literature suggests that personality can change throughout the life course, and such changes can provide valuable information in understanding and predicting important life outcomes. In the current study, we focus on change in contextualized personality, namely school extraversion, and its impact on cross-cultural adjustment. Compared to global traits, which cannot fully account for situational variations (Mischel, 1968, 1973; Wright and Mischel, 1987), contextualized personality captures one’s behavioral expressions of trait personality within a particular context (e.g., at work; Heller et al., 2009). For example, work contextualized personality captures one’s behavioral expressions of trait personality at work (Heller et al., 2009). In a similar vein, school extraversion represents the summary of a student’s extraverted behavior in the school context. Contextualized personality has been shown to outperform global traits in predicting context-specific outcomes (Schmit et al., 1995; Hunthausen et al., 2003; Bing et al., 2004). For instance, in a sample of 89 middle-aged women, Roberts and Donahue (1994) showed that contextualized personality varied across different roles (e.g., as a mother or as a worker) and role-specific personality had a significant advantage in predicting role-specific criteria in the corresponding context, whereas general personality yielded better prediction on general life outcomes. In a meta-analysis, Shaffer and Postlethwaite (2012) concluded that contextualized personality measures had higher validity in predicting job performance than global (i.e., non-contextualized) personality measures, such that the increases in validity exceeded at least 100% for four of the Big Five dimensions (openness to experience, extraversion, agreeableness, and emotional stability).

Given the proximity of contextualized personality, we intend to bridge the two areas of research (i.e., personality change and contextualized personality) and examine contextualized extraversion change and its relationship to cross-cultural adjustment outcomes. There are two major reasons for the use of this particular approach. First, personality change may be better captured in particular contexts, such that contextualized extraversion would experience more change than global extraversion. From a personality development perspective, evidence has supported the interaction between traits and contexts in shaping personality changes (Fraley and Roberts, 2005). Given the dynamics between the person and the environment, it is important to consider the corresponding context in which personality change may take place. From another point of view, life experiences and events can also lead to subsequent personality changes. For instance, Lüdtke et al. (2011) found that different life paths predicted different changes in personality, such that individuals on a vocational career path had higher increases in conscientiousness and lower increases in agreeableness than their counterparts who chose to pursue college degrees. In such cases, differences in contexts (vocation vs. school) may provide different cues that facilitate changes in personality. Therefore, as Lewis (1999) argued, the best way to study personality change is to examine behavior in context.

Second, contextualized personality change may provide better prediction than global personality change in the corresponding context. Bing et al. (2004) argued that the specificity of the reference point may account for the incremental validities of contextualized personality over global traits. Similarly, the validity of personality changes in predicting outcomes (in a particular context) may be improved by applying a specific context that responds with the criteria. Namely, specification of the context (e.g., school) may yield better and more accurate predictions in context-related outcomes (e.g., school satisfaction) due to the proximity of the predictors (e.g., school extraversion). Therefore, we focused on school extraversion and linked its initial level and change to cross-cultural adjustment outcomes.

*Hypothesis 1*: The initial levels of school extraversion will positively predict cross-cultural adjustment, such that students with higher initial school extraversion will have (a) better cross-cultural adjustment; (b) greater school satisfaction; and (c) lower withdrawal cognitions.
*Hypothesis 2*: Change in school extraversion will positively predict cross-cultural adjustment outcomes, such that increases in school extraversion will predict (a) better cross-cultural adjustment; (b) greater school satisfaction; and (c) lower withdrawal cognitions.

In an attempt to identify antecedents for personality change, we discuss the potential influence of global extraversion and cross-cultural motivation on the initial level and change in school extraversion. As the initial status of school extraversion represents how extraverted an individual is upon first assessment, we expect that global extraversion will likely be positively related to the initial level in school extraversion (see Bing et al., 2004; Heller et al., 2009). The impact of global extraversion on the change in school extraversion, however, is less clear. On the one hand, individuals who are already extraverted in general may be more prone to engage in and enjoy social interactions at school, and the positive feedback and experience in the overall “extraverted” environment may in turn prompt them to further elevate their extraversion in a school setting. On the other hand, introverts may have a greater potential than extraverts to increase their school extraversion in the process of cross-cultural adjustment because they have more room to grow.

*Hypothesis 3*: Global extraversion (Time 1) will positively predict the initial levels of school extraversion.
*Research Question 1*: How will global extraversion influence the slope of change in school extraversion?

Individuals with high cross-cultural motivation will have higher capacity and motives in learning and monitoring behaviors in a foreign context, which may result in them acting more extraverted in the US society. As described earlier, the US is among the most extraverted societies in the world, and sojourners coming from other cultures may encounter individuals who are more extraverted than those they are accustomed to interacting with. When these differences emerge, high cross-cultural motivation can trigger self-efficacy and help channel attention and effort in learning and adapting to the differences. As a result, when interacting with a group of extraverts, sojourners with high cross-cultural motivation may be more likely to develop strategies, such as acting more extraverted, in order to ensure smooth interactions and effective communication. In addition to being more self-efficacious, sojourners with high cross-cultural motivation also tend to show more intrinsic interests in learning and engaging in the cross-cultural experiences. Compared to people with low cross-cultural motivation, highly motivated individuals may be more likely to initiate interactions with the local people, learning more about the American culture, and be more motivated in adjusting behaviors (e.g., act more extraverted) in order to tackle the barriers in social interactions.

*Hypothesis 4*: Cross-cultural motivation (Time 1) will positively predict the initial levels of school extraversion.
*Hypothesis 5*: Cross-cultural motivation (Time 1) will positively predict the slope of change in school extraversion.

Thus far, our hypotheses are focused on extraversion as a higher-order factor of personality. According to DeYoung et al. (2007), extraversion encompasses two aspects, namely enthusiasm (positive emotion and sociability) and assertiveness (social dominance and the enjoyment of exhibitionism and leadership roles). In an exploratory fashion, we examined whether the pattern of results differ across these two aspects of extraversion.

## Materials and Methods

This research was approved by the Institutional Review Board at Wayne State University. Online informed consent was obtained from all participants in this research.

### Participants

The current sample consisted of first-year undergraduate and graduate international students from sixteen universities who had recently arrived at the United States at the time of the first survey. To maximize the representativeness of the current sample, two methods were utilized for recruitment: (1) An e-mail advertisement about the study was sent to the Office of International Students and Scholars of 157 universities across the United States to solicit eligible international students. In order to reach out to universities across the United States with relatively large international student bodies, the names of the universities were obtained from (1) the list of National Universities with Most International Students on USNews (USNews, 2012), and (2) the list of Accredited Programs in Clinical Psychology on the official website of American Psychological Association (American Psychological Association [APA], 2012). Thirteen universities (response rate = 8.28%) agreed to advertise the current survey via Listserv or Newsletter; and (3) a Facebook message was sent to 27 universities who had a Facebook page and did not respond to the e-mail inquiry. Two universities (response rate = 7.40%) agreed to post a study advertisement on their official Facebook page. To ensure that the current sample consists of only newly arrived international students, we screened out students who were not from a foreign country or had been in the US for 3 months or longer.

Two hundred and eighty-nine individuals provided sufficient data (i.e., no missing values on global extraversion or cross-cultural motivation) to be included in the analysis (57% males; average age = 23, *SD* = 5). Participants were asked to complete three questionnaires over 4 months after arriving in the United States. Participants in the current study reported coming from a diverse range of countries, with the top five countries being China (22%), Canada (13%), Australia (8%), Japan (7%), and India (6%).

### Procedure

Acknowledging the limitation of cross-sectional designs in making predictive inferences, the present study adopted a longitudinal design to better capture the changes in personality and to make a stronger test for predictive values of such changes (Funder, 2008). Pinpointing the timeframe for longitudinal changes to occur can be challenging, as the patterns of change can depend on a multitude of environmental and personal factors in the transition process. However, it has been shown that most issues related to adaptation occur in the early stage of a cross-cultural experience (Ward et al., 1998; Ying, 2005). Therefore, in the current study, cross-cultural adjustment was captured in the first 4 months (approximately one academic semester) after international students’ arrival in the states. Specifically, participants were asked to complete measures of school extraversion, global extraversion, and cross-cultural motivation within the first month after arriving in the US (Time 1), followed by the second assessment of school extraversion 2 months after the first wave (Time 2) and the third assessment of school extraversion together with cross-cultural adjustment outcomes (i.e., cross-cultural adjustment, withdrawal cognitions, and school satisfaction) 4 months after the first wave (Time 3). Participants who completed the study were compensated a $10 gift card and a chance to win a $50 gift card based on random drawing.

### Screening for Insufficient Effort Responding

To ensure data quality, we utilized two measures of insufficient effort responding (IER; Huang et al., 2012) to screen participants who did not fully attend to the survey instructions and items (see DeSimone et al., 2015). Removing IER prior to data analysis is important because IER can have potential deleterious impact on survey results (Huang et al., 2014b). First, we used three items from a validated infrequency scale (Huang et al., 2014a) designed to detect IER in a low-stakes survey context. The three items presented counterfactual statements (i.e., “I have never used a computer”; “Eat cement occasionally”; and “Can teleport across time and space.”) where deviation from choosing the “correct” answers would indicate possible IER behavior. The three items were scattered in the first survey. Any response option indicating disagreement to the counterfactual statements was coded as attentive responding (0), whereas the other response options were coded as IER (1). The IER scale score was computed as the average of the three dichotomized item scores (α = 0.75).

The second operationalization of IER used the response time approach, where an unrealistically short survey completion time was used to indicate IER. We adopted Huang et al.’s (2012) 2 s per item criterion and flagged individuals who responded faster than this on each survey. Participants were coded as IER (1) based on survey completion time if they sped through at least one of the three surveys and attentive responding (0) if otherwise.

To maximally retain the sample and avoid misclassifying attentive respondents, we followed Huang et al.’s (2012) recommendation to remove respondents who clearly engaged in IER behavior. Specifically, we excluded responses that (a) scored 1 on the IER scale (i.e., failing all three IER items); and (b) sped through at least one of the surveys. This *post hoc* decision rule was made after the data collection but before testing the current research model. Out of the 289 participants, 74 (25.61%) individuals were flagged and removed from subsequent analyses, leaving the final sample of 215 participants (54% male; *M*_age_ = 24 years, *SD* = 4).

### Measures

#### Global Extraversion

Global extraversion (α = 0.77) was measured with the 20-item extraversion scale from DeYoung et al. (2007), which measures two aspects of extraversion, namely enthusiasm (α = 0.73) and assertiveness (α = 0.65). Participants were asked to rate how well each item accurately described themselves. Sample items include: “Make friends easily” (enthusiasm) and “Take charge” (assertiveness). All items were administered on a seven-point Likert scale, ranging from 1 (very inaccurate) to 7 (very accurate).

#### School Extraversion

To assess School extraversion, we adapted the global extraversion measure by asking participants to reflect only on their behavior at school settings (see Schmit et al., 1995; Bing et al., 2004). All items were administered on a seven-point Likert scale, ranging from 1 (very inaccurate) to 7 (very accurate). Cronbach’s alphas were 0.84, 0.77, and 0.72 across the three waves, respectively. For the two aspects of school extraversion, the Cronbach’s alphas were 0.79, 0.76, and 0.71 for school enthusiasm and 0.71, 0.68, and 0.63 for school assertiveness, respectively.

#### Cross-cultural Motivation

Ang et al.’s (2007) five-item motivational cultural intelligence (CQ) scale was used to assess cross-cultural motivation (see Chen et al., 2010). This measure captures both cross-cultural self-efficacy (a sample item is: “I am confident that I can socialize with locals in a culture that is unfamiliar to me.”) and cross-cultural intrinsic motivation (a sample item is: “I enjoy interacting with people from different cultures.”). All items were administered on a seven-point Likert scale, ranging from 1 (strong disagree) to 7 (strongly agree). Cronbach’s alpha of the scale was 0.82.

#### Cross-cultural Adjustment

We used a 14-item scale from Black (1988) to assess three facets of cross-cultural adjustment: general (seven items; α = 0.83), interaction (four items; α = 0.86), and work (three items; α = 0.80). Items pertaining to work adjustment were adapted to the school context. Participants were asked the extent to which they feel adjusted (or unadjusted) to the various aspects of their life in the US. All items were administered on a seven-point Likert scale, ranging from 1 (very unadjusted) to 7 (very adjusted).

#### Withdrawal Cognitions

Withdrawal cognitions were measured based on Shaffer et al.’s (2006) six-item scale (adapted from Hom and Griffeth, 1991). A sample item is: “I plan to leave this school.” All items were administered on a five-point Likert scale, ranging from 1 (strong disagree) to 5 (strongly agree). The measure had a Cronbach’s alpha of 0.97.

#### School Satisfaction

Satisfaction was assessed in the school context using a seven-item scale from Lounsbury et al. (2005). A sample questions is “How satisfied are you with how much you are leaning in school?” All items were administered on a seven-point Likert scale, ranging from 1 (very dissatisfied) to 7 (very satisfied). Cronbach’s alpha was 0.83 for the scale.

## Results

Descriptive statistics and zero-order correlations are presented in **Table 1.** Global extraversion positively correlated with school extraversion assessed at each time point (*r*s = 0.77, 0.55, and 0.54, respectively, *p* < 0.001), and the estimates were in line with the correlations previous reported between global and contextualized personality (e.g., Bing et al., 2004; Heller et al., 2009). Global extraversion (Time 1) and school extraversion at each time point was positively associated with the three aspects of cross-cultural adjustment and school satisfaction but was unrelated to withdrawal cognitions.

**Table 1:** Descriptive statistics and intercorrelations for study variables.

	Survey Scale	1	2	3	4	5	6	7	8	9	10	11	12	13	14	15	16
1	T1 global extraversion																


2	T1 school extraversion	0.77^∗∗∗^															


3	T1 school enthusiasm	0.73^∗∗∗^	0.89^∗∗∗^														


4	T1 school assertiveness	0.61^∗∗∗^	0.86^∗∗∗^	0.54^∗∗∗^													


5	T1 cross-cultural motivation	0.51^∗∗∗^	0.46^∗∗∗^	0.41^∗∗∗^	0.39^∗∗∗^												


6	T2 school extraversion	0.52^∗∗∗^	0.55^∗∗∗^	0.48^∗∗∗^	0.45^∗∗∗^	0.32^∗∗∗^											


7	T2 school enthusiasm	0.50^∗∗∗^	0.49^∗∗∗^	0.53^∗∗∗^	0.30^∗∗∗^	0.39^∗∗∗^	0.79^∗∗∗^										


8	T2 school assertiveness	0.32^∗∗∗^	0.36^∗∗∗^	0.22^∗∗^	0.41^∗∗∗^	0.11	0.78^∗∗∗^	0.23^∗∗^									


9	T3 school extraversion	0.51^∗∗∗^	0.54^∗∗∗^	0.44^∗∗∗^	0.50^∗∗∗^	0.35^∗∗∗^	0.69^∗∗∗^	0.54^∗∗∗^	0.54^∗∗∗^								


10	T3 school enthusiasm	0.45^∗∗∗^	0.43^∗∗∗^	0.45^∗∗∗^	0.30^∗∗∗^	0.33^∗∗∗^	0.62^∗∗∗^	0.61^∗∗∗^	0.36^∗∗∗^	0.81^∗∗∗^							


11	T3 school assertiveness	0.35^∗∗∗^	0.41^∗∗∗^	0.23^∗∗^	0.50^∗∗∗^	0.22^∗∗^	0.47^∗∗∗^	0.23^∗∗^	0.51^∗∗∗^	0.77^∗∗∗^	0.25^∗∗∗^						


12	T3 general adjustment	0.27^∗∗∗^	0.31^∗∗∗^	0.32^∗∗∗^	0.21^∗∗^	0.51^∗∗∗^	0.37^∗∗∗^	0.47^∗∗∗^	0.10	0.31^∗∗∗^	0.29^∗∗∗^	0.19^∗∗^					


13	T3 interaction adjustment	0.28^∗∗∗^	0.28^∗∗∗^	0.30^∗∗∗^	0.17^∗^	0.55^∗∗∗^	0.25^∗∗∗^	0.35^∗∗∗^	0.04	0.25^∗∗∗^	0.24^∗∗∗^	0.14^∗^	0.69^∗∗∗^				


14	T3 school adjustment	0.22^∗∗∗^	0.28^∗∗∗^	0.32^∗∗∗^	0.16^∗^	0.39^∗∗∗^	0.26^∗∗∗^	0.34^∗∗∗^	0.06	0.27^∗∗∗^	0.26^∗∗∗^	0.15^∗^	0.78^∗∗∗^	0.62^∗∗∗^			


15	T3 school satisfaction	0.39^∗∗∗^	0.31^∗∗∗^	0.32^∗∗∗^	0.22^∗∗^	0.61^∗∗∗^	0.39^∗∗∗^	0.45^∗∗∗^	0.16^∗^	0.24^∗∗∗^	0.24^∗∗∗^	0.13	0.52^∗∗∗^	0.50^∗∗∗^	0.46^∗∗∗^		


16	T3 withdrawal cognitions	0.03	0.03	-0.01	0.07	0.35^∗∗∗^	-0.11	0.01	-0.18^∗^	-0.08	-0.07	-0.07	0.30^∗∗∗^	0.31^∗∗∗^	0.27^∗∗∗^	0.24^∗∗∗^	


	*M*	4.43	4.33	4.31	4.34	5.56	4.37	4.49	4.26	4.35	4.40	4.29	5.26	5.12	5.38	5.34	3.90


	*SD*	0.66	0.75	0.91	0.79	0.89	0.62	0.80	0.78	0.60	0.78	0.73	0.87	1.08	0.99	0.83	2.05




*N = 211–215. ^∗^p < 0.05; ^∗∗^p < 0.01; ^∗∗∗^p < 0.001.*

Prior to hypothesis testing, we examined whether school extraversion at the initial time point added incremental validity above and beyond global extraversion in predicting cross-cultural adjustment outcomes. Without controlling for school extraversion, global extraversion predicted the three aspects of cross-cultural adjustment (general adjustment, β = 0.31, *p* < 0.001; interaction adjustment, β = 0.31, *p* < 0.001; school adjustment, β = 0.25, *p* < 0.001) and school satisfaction (β = 0.39, *p* < 0.001), but not withdrawal cognitions (β = 0.03, *p* = 0.68). To test the incremental validity of school extraversion above and beyond global extraversion, we used hierarchical multiple regression and regressed the cross-cultural adjustment outcome variables separately onto global extraversion (step 1) and school extraversion (step 2). Results revealed that school extraversion significantly predicted school adjustment when controlling for global extraversion, β = 0.22, *p* = 0.04, explaining 2% additional variance. However, school extraversion did not add any significant incremental prediction for any of the other outcomes.

We employed latent growth modeling (LGM; Chan, 2002) to test the hypotheses. Based on structural equation modeling, LGM allows for modeling and estimating different parameters of change in a longitudinal dataset (Lance et al., 2000; Chan, 2002). Specifically, we used two latent factors (Kline, 2005; Kaplan, 2009; e.g., Chan and Schmitt, 2000) to model the change trajectory for school extraversion: (a) the latent intercept factor that represents the initial status of school extraversion (i.e., how extraverted an individual is upon first assessment); and (b) the latent slope factor that represents the rate of change in school extraversion (i.e., how an individual’s extraversion has changed across the span of the study). In light of the present sample size, we used observed scale scores as indicators in the LGM analysis.

**FIGURE 2:**
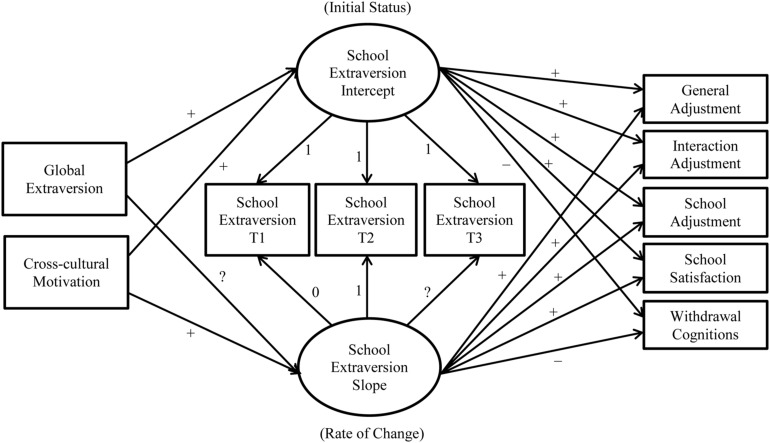
Model 2 with hypotheses.

^∗^*p* < 0.05; ^∗∗^*p* < 0.01; ^∗∗∗^*p* < 0.001; ^†^*p* ≥ 0.05; *N* = 215. Estimates are standardized estimates from the modified Model 1 (i.e., the constrained model). Solid lines represent significant paths, whereas dotted lines represent insignificant paths.

We tested two nested models to assess whether there was non-linear change in school extraversion. For the intercept term, both models fixed factor loadings to one for each of the three school extraversion measures. In contrast, for the slope term, the initial constrained model (Model 1, see **Figure 1**) fixed factor loadings to 0, 1, and 2 for T1–T3 school extraversion, respectively, whereas the unconstrained model (Model 2, see **Figure 2**) fixed factor loadings for T1 and T2 school extraversion but freely estimated the loading for T3 school extraversion. Thus, the intercept term indicates the initial level of school extraversion, while the slope term indicates the rate of change in school extraversion. In both models, we included global extraversion and cross-cultural motivation as predictors for the intercept and slope of school extraversion.

**FIGURE 3:**
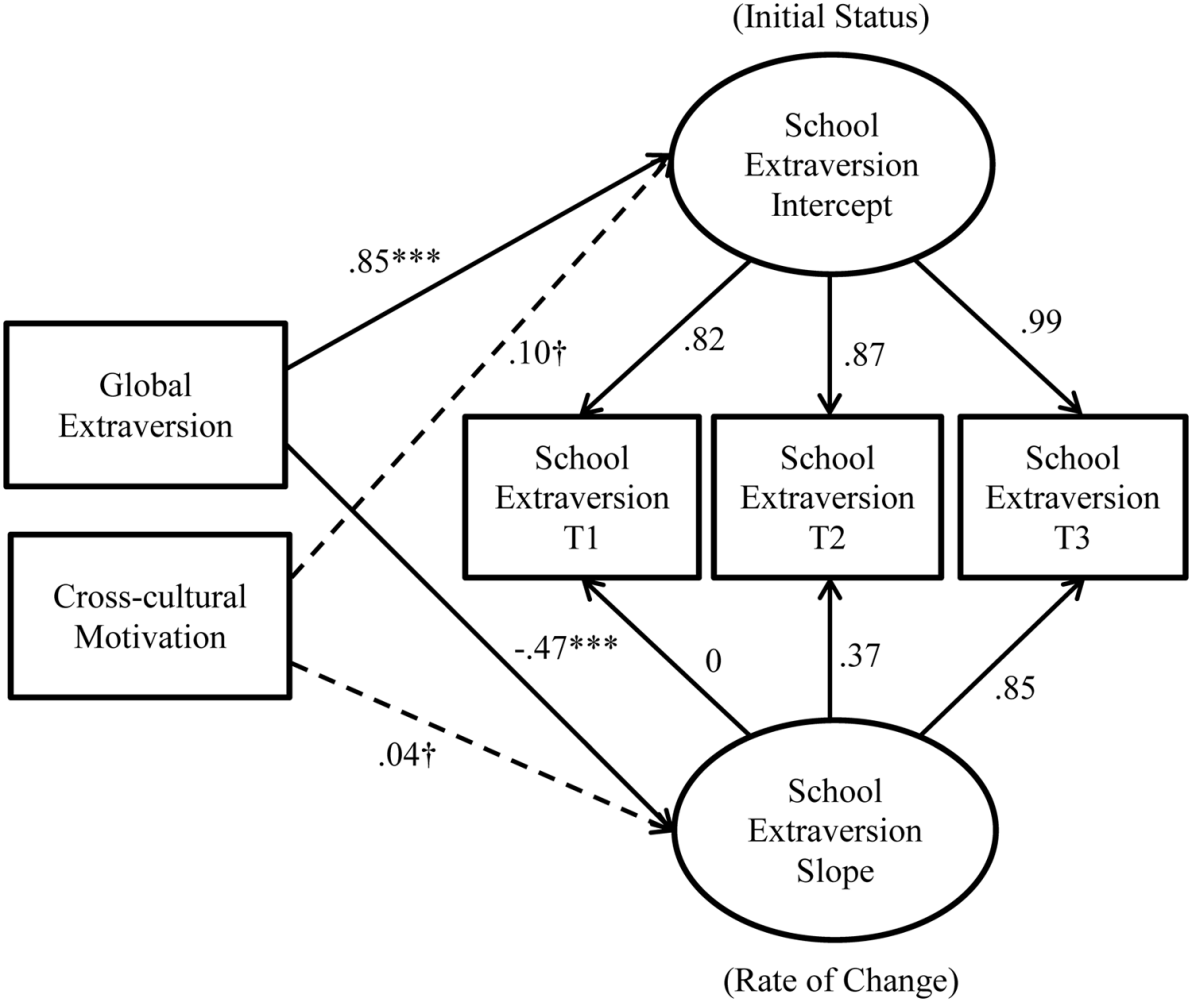
Results from the modified Model 1 with the predictors and school extraversion change.

**FIGURE 4:**
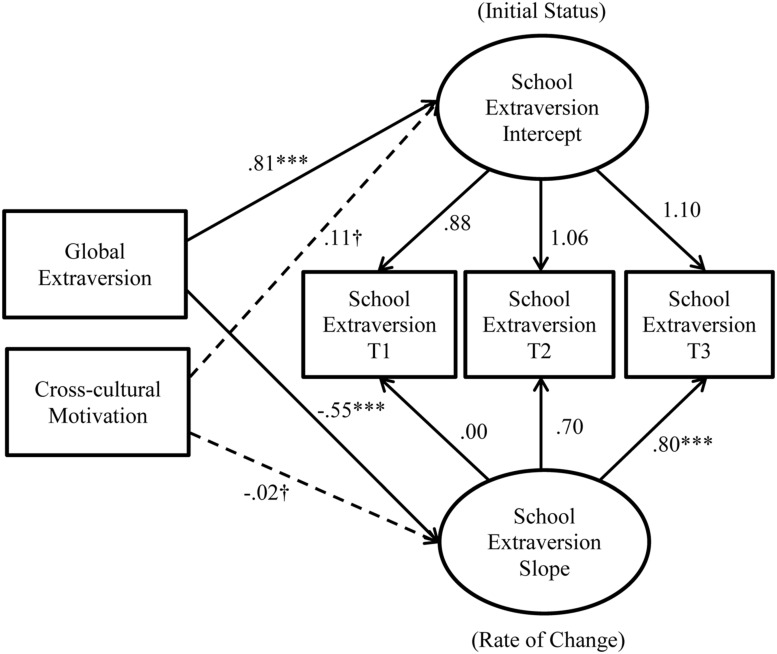
**Results from the modified Model 2 with the predictors and school extraversion change.** ^∗^*p* < 0.05; ^∗∗^*p* < 0.01; ^∗∗∗^*p* < 0.001; ^†^*p* ≥ 0.05; *N* = 215. Estimates are standardized estimates from the modified Model 2 (i.e., the unconstrained model). Solid lines represent significant paths, whereas dotted lines represent insignificant paths.

Initial model testing revealed that Models 1 and 2 did not converge because a correlation estimate went outside of the reasonable bounds. Specifically, the correlation between cross-cultural motivation and the slope of school extraversion change was estimated to be -1.01 for Model 1 and -1.15 for Model 2, respectively. Given this error, we started diagnosing the cause of this issue by removing the adjustment outcomes from Models 1 and 2 to focus on the effects of the predictors (i.e., global extraversion and cross-cultural motivation) on school extraversion change (intercept and slope). These simplified models converged reasonably well: modified Model 1 (see **Figure 3**): χ^2^(3) = 12.52, CFI = 0.98, RMSEA = 0.12, SRMR = 0.16; modified Model 2 (see **Figure 4**): χ^2^(2) = 1.44, CFI = 1.00, RMSEA = 0.00, SRMR = 0.01. Importantly, cross-cultural motivation did not predict either the intercept or the slope of school extraversion change (Model 1: βs = 0.10 and 0.04, respectively; Model 2: βs = 0.11 and -0.02, respectively). These non-significant paths were incongruent with the observed strength of relationships between cross-cultural motivation and adjustment outcomes (see **Table 1**), suggesting that the erroneous estimates were caused by the fact that the influence of cross-cultural motivation on adjustment outcomes could not be accounted for in Models 1 and 2. In addition, a chi-square difference test revealed that relaxing the constraint on the slope significantly improved the model fit, Δχ^2^(1) = 11.08, *p* < 0.001, suggesting that the change was non-linear (thus retaining Model 2).

**FIGURE 5:**
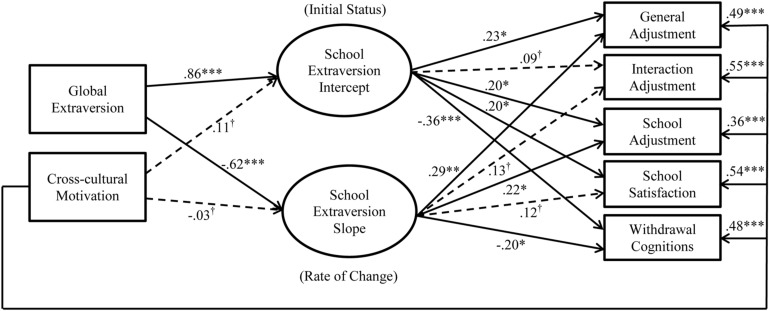
**Results from Model 2 with direct paths from cross-cultural motivation to cross-cultural adjustment outcomes.** ^∗^*p* < 0.05; ^∗∗^*p* < 0.01; ^∗∗∗^*p* < 0.001; ^†^*p* ≥ 0.05; *N* = 215. Estimates are standardized estimates from Model 2 (i.e., the unconstrained model). Observed measures of school extraversion were included in the model but not presented here. Solid lines represent significant paths, whereas dotted lines represent insignificant paths.

Given past research that suggests a positive influence of cross-cultural motivation on cross-cultural adjustment and adaptation (e.g., Templer et al., 2006; Ang et al., 2007; Chen et al., 2010), we reestimated Model 2 with direct paths from cross-cultural motivation to cross-cultural adjustment outcomes. In other words, in these reestimated models, cross-cultural motivation served the role of a covariate such that its influence on adjustment outcomes could be controlled for. The model had reasonably good fit to the data (see **Figure 5**): χ^2^(13) = 26.32, CFI = 0.99, RMSEA = 0.07, SRMR = 0.03. The loading for the slope on T3 school extraversion was 0.98, indicating a nearly flat rate of change from T2 to T3 on school extraversion.

We proceeded to examine the hypotheses with the estimates from Model 2 (see **Figure 5**). The initial status of school extraversion predicted two aspects of cross-cultural adjustment (general adjustment, β = 0.23, *p* = 0.01; school adjustment, β = 0.20, *p* = 0.03), school satisfaction (β = 0.20, *p* = 0.03), and withdrawal cognitions (β = -0.36, *p* < 0.001), but not interaction adjustment (β = 0.09, *p* = 0.27). Therefore, *Hypotheses 1a* was partially supported, while *Hypotheses 1b* and *1c* were fully supported. Meanwhile, the slope of change in school extraversion predicted two aspects of cross-cultural adjustment (general adjustment, β = 0.29, *p* = 0.002; school adjustment, β = 0.22, *p* = 0.02) and withdrawal cognitions (β = -0.20, *p* = 0.04), but not interaction adjustment (β = 0.13, *p* = 0.13) or school satisfaction (β = 0.12, *p* = 0.18). Thus, *Hypothesis 2a* was partially supported, *Hypothesis 2b* was not supported, and *Hypothesis 2c* was fully supported.

**FIGURE 6:**
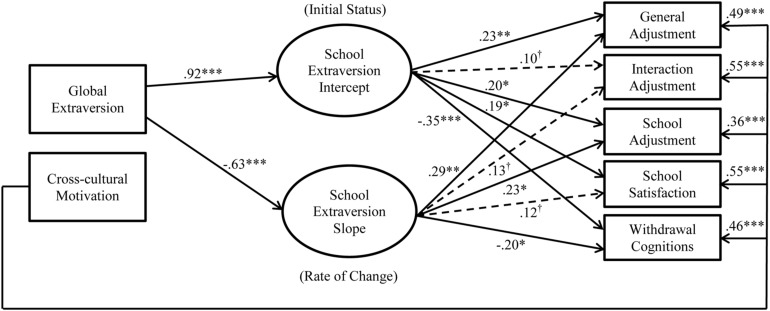
**Results from the final Model 2 omitting the paths from cross-cultural motivation to change in school extraversion.** ^∗^*p* < 0.05; ^∗∗^*p* < 0.01; ^∗∗∗^*p* < 0.001; ^†^*p* ≥ 0.05. *N* = 215. Estimates are standardized estimates from Model 2 (i.e., the unconstrained model). Observed measures of school extraversion were included in the model but not presented here. Solid lines represent significant paths, whereas dotted lines represent insignificant paths.

Supporting *Hypothesis 3*, global extraversion (β = 0.86, *p* < 0.001) significantly and positively predicted the intercept of school extraversion. Interestingly, global extraversion *negatively* predicted the slope of change in school extraversion (β = -0.62, *p* < 0.001). Furthermore, cross-cultural motivation did not predict the intercept (β = 0.11, *p* = 0.06) or the slope of change in school extraversion (β = -0.03, *p* = 0.79), thus failing to support *Hypotheses 4* and *5*. For a comparison purpose, we tested Model 2 (i.e., the unconstrained model) with the full sample (*N* = 289) without excluding any cases marked as IER. The model had good fit: χ^2^(13) = 25.46, CFI = 0.99, RMSEA = 0.06, SRMR = 0.02. The directions and the significance of the paths were similar to those in the IER-excluded sample (*N* = 215), except that the intercept of school extraversion no longer predicted school adjustment, β = 0.14, *p* = 0.06. Based on these results, we retained a more simplified model excluding the non-significant paths from cross-cultural motivation to change in school extraversion (see **Figure 6**). It should be noted that the model trimming at this stage did not affect the conclusions regarding the substantive effects hypothesized above.

Given that our model suggests a potential mediating effect of school extraversion (both in terms of initial status and rate of change) on the relationship between global extraversion and cross-cultural adjustment outcomes, we tested a partial mediation model by allowing the direct paths from global extraversion to the cross-cultural adjustment outcomes [model fit: χ^2^(8) = 18.95, CFI = 0.99, RMSEA = 0.08, SRMR = 0.05], and compared it with Model 2. A chi-square difference test revealed that adding the direct paths did not significantly improve the model fit, Δχ^2^(5) = 7.37, *p* = 0.19, indicating that the more parsimonious Model 2 should be retained. In addition, results from the partial mediation model showed that none of the paths from global extraversion to cross-cultural adjustment outcomes was significant, while the paths from global extraversion to change in school extraversion and from change in school extraversion to adjustment outcomes all remained similar to those in Model 2. This pattern of results indicates that global extraversion does not directly impact cross-cultural adjustment outcomes, but rather influences them through the initial level and rate of change in school extraversion.

In an exploratory fashion, we examined the two aspects of school extraversion (i.e., enthusiasm and assertiveness) separately to see if either of them was driving the results. Specifically, we retested Model 2 using school enthusiasm (Model 3) and school assertiveness (Model 4) separately. Results from Model 3 showed that there was a significant mean level increase in school enthusiasm (*M*s = 0.17, *p* = 0.004). The initial status of school enthusiasm predicted all three aspects of cross-cultural adjustment (general adjustment, β = 0.31, *p* = 0.001; interaction adjustment, β = 0.17, *p* = 0.04; school adjustment, β = 0.28, *p* = 0.03), school satisfaction (β = 0.26, *p* = 0.001), and withdrawal cognitions (β = -0.29, *p* = 0.002). Meanwhile, the slope of change in school enthusiasm also predicted the three aspects of cross-cultural adjustment (general adjustment, β = 0.38, *p* < 0.001; interaction adjustment, β = 0.22, *p* = 0.02; school adjustment, β = 0.29, *p* = 0.006) and school satisfaction (β = 0.21, *p* = 0.01), but not withdrawal cognitions (β = -0.08, *p* = 0.42).

In terms of school assertiveness, results from Model 4 showed that there was not a significant mean level change in school assertiveness (*M*s = -0.08, *p* = 0.18). In addition, the initial status of change in school assertiveness negatively predicted withdrawal cognitions (β = -0.27, *p* = 0.002) but did not predict any of the three aspects of cross-cultural adjustment (general adjustment, β = 0.07, *p* = 0.43; interaction adjustment, β = -0.02, *p* = 0.79; school adjustment, β = 0.05, *p* = 0.03) or school satisfaction (β = 0.06, *p* = 0.45). Likewise, the slope of change in school assertiveness did not predict the three aspects of cross-cultural adjustment (general adjustment, β = 0.11, *p* = 0.34; interaction adjustment, β = 0.02, *p* = 0.89; school adjustment, β = 0.07, *p* = 0.59), school satisfaction (β = -0.03, *p* = 0.81), or withdrawal cognitions (β = -0.12, *p* = 0.42). Therefore, we conclude that school enthusiasm, but not school assertiveness, was the driving force behind school extraversion change and cross-cultural adjustment.

## Discussion

Recognizing the malleable aspect of personality and its potential beneficial effect on cross-cultural adjustment in the US, the current study marks a first attempt to capture the *process* by which sojourners experience changes in personality during cross-cultural adaptation, while positioning the changes as proximal antecedents to cross-cultural adjustment outcomes. As expected, students with higher initial school extraversion also had better cross-cultural adjustment in general and at school, greater school satisfaction, and lower withdrawal cognitions. More importantly, increases in school extraversion were shown to positively predict general and school-specific adjustment and negatively predict withdrawal cognitions. In addition, global extraversion positively predicted the initial level of school extraversion yet negatively predicted the rate of change in school extraversion. Findings from the current study lay the ground work for investigating personality changes in specific contexts pertaining to work-related transitions and adaptation. Taken together, this study makes a number of notable contributions to the literatures of cross-cultural adjustment and personality and sheds light on practices in sojourners and expatriate assessment and selection.

### Theoretical and Practical Implications

In line with the cross-cultural adjustment literature (e.g., Swagler and Jome, 2005; Sri Ramalu et al., 2010), the current study highlights the important role of contextualized extraversion in facilitating sojourners’ adjustment in a new cultural environment. Our findings demonstrate that school extraversion and its change predicted both general cross-cultural adjustment and academic related adjustment (i.e., school adjustment, withdrawal cognitions). The positive effects of school extraversion and its change on general and school adjustment in the US might be attributed in part to the overall high level of extraversion in the US society (see McCrae and Terracciano, 2005), such that sojourners who are more extraverted or becoming more extraverted in the school context may find their own characteristics increasingly congruent with those of the cultural environment, which in turn lead to better adjustment outcomes. Although in the expected directions, neither the initial status nor the change in school extraversion predicted interaction adjustment, suggesting that extraversion in the school context may not have a direct impact on international students’ socialization and interaction with the host nationals in general. That is, as interaction with host nationals can occur beyond the school context, extraversion and its changes contextualized within the school context may not be particularly fitting to predict interaction adjustment outside of the school context. In addition, exploratory analyses on the two aspects of school extraversion (i.e., enthusiasm and assertiveness) showed that school enthusiasm, but not school assertiveness, was the driving force behind school extraversion change and cross-cultural adjustment.

Our findings imply that entering and living in a new culture can lead to changes in one’s contextualized personality, a notion that is in line with the socialization effects (Roberts and Mroczek, 2008; Roberts et al., 2008). On the other hand, one should consider the potential selection effects (Headey and Wearing, 1989; Roberts and Wood, 2006) simultaneously. For instance, individuals who choose to study or work abroad may tend to be more extraverted than those who do not. Likewise, international students who are more extraverted or becoming more extraverted in the school context may also select themselves or be selected into more desirable and acceptable events (e.g., study groups, social activities) that may facilitate their adaptation. Future research should test these factors as potential mechanisms via which personality and its change exert effects on cross-cultural adjustment.

Building upon the previous research that has demonstrated the proximity of contextualized personality, we posit that personality change may be better captured in particular contexts, and that contextualized personality change may provide better prediction than global personality change in the corresponding context. Therefore, we examined extraversion changes in a specific context, the school context, and linked these changes to cross-cultural adjustment outcomes. In doing so, the current study advances the existing literature that has been mainly focused on the contextualization of stable personality traits and shows promising results of using contextualized personality changes to predict context specific and general outcomes.

In an attempt to find out what drives contextualized personality change, we examined the relations of two individual differences (i.e., global extraversion and cross-cultural motivation) with school extraversion change. We found that global extraversion, although positively related to the initial status of school extraversion, negatively predicted the rate of change in school extraversion. In other words, international students who had higher global extraversion also tended to behave more extraverted at school in the beginning but were also more likely to experience a decrease in school extraversion, whereas individuals who had lower global extraversion also behaved more introverted at school in the beginning but may experience an increase in school extraversion. This suggests that introverts may have a greater potential and more room than extraverts to increase their contextualized extraversion while adjusting to a new culture. Although global extraversion was inversely related to the slope of change in school extraversion, descriptive statistics indicate that individuals who were high on global extraversion remained more extraverted in school (5.21 at T1 to 4.74 at T3) compared to those who were low on global extraversion (3.56 at T1 to 3.90 at T3), despite the changes.

Proposing that individuals with high cross-cultural motivation will have high capacity and motivation to behave extraverted in the US, we examined cross-cultural motivation as an antecedent for the initial status and change in school extraversion. Failing to support our hypothesis, we did not find any evidence linking cross-cultural motivation to either the initial status or change in school extraversion. Therefore, the current study’s findings suggest that school extraversion and its change may be more driven by one’s standing on global extraversion than by cross-cultural motivation.

Despite the growing interest in studying cross-cultural adjustment, the current study is the first to integrate the recent developments in personality research in a cross-cultural context. Based on the findings, the current research echoes past studies (e.g., Caligiuri, 2000) that suggest organizations in the US might incorporate extraversion as a selection tool for sojourners and expatriate workers. Meanwhile, practitioners should recognize the potential malleability of contextualized personality, especially under the influence of other individual characteristics (e.g., global personality), and how personality change may predict adjustment and withdrawal. By identifying changes in contextualized extraversion and their impact on cross-cultural adjustment, the current study broadens our understanding of the role of personality in adaptation and adjustment. Particularly, stable personality traits have been examined to understand how individuals adapt to changes in their work environment (Huang et al., 2014c). On the other end of the continuum, individuals respond to changes in their task context with varying personality states (Minbashian et al., 2010; Huang and Ryan, 2011). The current study identified contextualized extraversion change as an additional mechanism, beyond stable traits and momentary states, that may contribute to one’s acceptance of environmental and organizational change, as well as psychological and work adjustment (e.g., adaptive performance, see Jundt et al., 2015).

### Limitations

Despite the contributions, this study has a few limitations. First, international students in the current study were facing dual challenges of adapting to a new culture and adjusting to college. Therefore, it is unclear to what extent personality change was driven by cultural influence or school experience. Future studies may further tease apart the cultural and school influences on personality change by measuring them separately or using a non-student sample.

Second, the current findings, based on international students, may not be readily applied to foreign workers adjusting to the US culture. Although international students may share similar encounters and experiences with organizational sojourners and expatriates in a foreign culture (e.g., cultural shock and adaptation), and that personality traits (e.g., conscientiousness) have been shown to predict similar outcomes in work and school contexts (e.g., job performance and school performance; Barrick and Mount, 1991; Bing et al., 2004), it is unknown whether findings from the current research can be fully replicated in organizational settings. Therefore, researchers are encouraged to replicate this study using organizational sojourners and expatriates.

Third, while we focused on extraversion in the current study given the cultural context, the other four Big Five traits (i.e., agreeableness, conscientiousness, openness to experience, and neuroticism) have also been linked to cross-cultural adjustment in other cultures (e.g., Swagler and Jome, 2005; Peltokorpi, 2008; Sri Ramalu et al., 2010). Although we attempted to explore changes in the other four personality dimensions by tracking them via the mini-IPIP scales (Donnellan et al., 2006), we were unable obtain reliable measures with four items on each personality dimension. Therefore, a venue for future research is to extend the current study and further examine the relationship between changes of other personality dimensions and cross-cultural adjustment.

### Future Directions

As the first study to examine contextualized personality change in the cross-cultural adjustment context, the current study points to a few interesting venues for future research. The first direction for future research pertains to individual factors that may contribute to personality changes in a cross-cultural context. In the current study, we examined global extraversion and cross-cultural motivation as two individual characteristics that may drive changes in school extraversion. Meanwhile, other individual differences may also lead one’s contextualized personality to change in a cross-cultural context. For instance, openness to experience may influence the extent to which one is susceptible to cultural influences and subsequently how he or she behaves in a cross-cultural context. Self-monitoring, the extent to which an individual observes and controls his or her behavior according to situational cues (Snyder, 1974), may shape how this person adjusts behaviors when encountered with a new cultural environment. Demographic variables, such as age, may also play a role in whether and how much personality changes during cross-cultural adaptation.

Second, future research should investigate personality changes in other important, specific contexts pertaining to work-related transitions and adaptation. Studying personality changes in different contexts (e.g., cultural, work, and family) is important because not only can it provide insight on the potential varying degrees of personality changes associated with particular contexts (e.g., personality changes may be more pronounced in a cross-cultural context than in an organizational socialization context), but it can also improve the predictive validity of the individual characteristics for the outcomes of interest. For instance, the model of attraction–selection–attrition (ASA; Schneider, 1987) indicates that organizational newcomers who share similar characteristics with the existing employees should be more likely to stay in the organization and less likely to withdraw. Considering the malleable aspect of contextualized personality, it is possible that some newcomers may experience changes in work contextualized personality that can enable them to fit better with the work group. Therefore, newcomer contextualized personality changes may predict newcomer adjustment and turnover during organizational socialization.

Third, despite the fact that personality and life experiences are interactive in nature, limited research has been conducted to study the two aspects in conjunction. As discussed earlier, the interplay of personality and life events can be referred to as selection effects and socialization effects. The current findings regarding changes in contextualized personality show support for the socialization effects (Roberts and Mroczek, 2008; Roberts et al., 2008). Extending the previous longitudinal studies that have demonstrated both the selection effects and the socialization effects (e.g., Vaidya et al., 2002; Specht et al., 2011), future research on personality changes in a cross-cultural context should examine the potential impact of cross-cultural adjustment outcomes on personality. For instance, a sojourner who is successfully adjusting to the American culture may also become more interested in reaching out to the local nationals, attending activities and events, and staying an active part of his or her surroundings, all of which indicate an increasing level of extraversion. In contrast, a sojourner who experiences difficulty in adapting to a new cultural environment may further withdraw from social interactions and activities, leading to a decrease in extraversion. Therefore, future research should explore the reciprocal relationship between personality and adaptation outcomes.

Fourth, provided the growing literature that suggests change in trait personality (e.g., Roberts et al., 2001; Vaidya et al., 2002; Lüdtke et al., 2011; Boyce et al., 2015), it would be an interesting research question to examine the potential change in trait extraversion in a cross-cultural context. It is likely that, given the prolonged influence of cross-cultural events and experiences, some individuals might eventually experience significant changes in their trait personality (e.g., become more extraverted in general). Although we did not examine change in trait extraversion in our study, the current findings regarding change in contextualized extraversion and its predictive validity lay the groundwork for studying trait changes in the future.

Fifth, based on the findings that contextualized personality may be malleable, organizational researchers and practitioners may explore the feasibility of developing training interventions that aim to elevate certain contextualized personality characteristics (cf. Huang and Ford, 2012) among sojourners and expatriates based on the cultural context.

## Conclusion

The current captures the *process* by which sojourners experience changes in personality during cross-cultural adaptation and examine how these changes relate to cross-cultural adjustment outcomes. By demonstrating that the initial status and change in school extraversion predict cross-cultural adjustment outcomes, our findings lay the ground work for investigating personality changes in specific contexts pertaining to work-related adaptation and shed light on practices in sojourner and expatriate assessment and selection.

## Conflict of Interest Statement

The authors declare that the research was conducted in the absence of any commercial or financial relationships that could be construed as a potential conflict of interest.

## References

[B1] American Psychological Association [APA] (2012). *Accredited Programs in Clinical Psychology.* Available at: http://www.apa.org/ed/accreditation/programs/index.aspx [accessed July 15 2012].

[B2] Anderson B. A. (2005). Expatriate selection: good management or good luck? *Int. J. Hum. Resour. Manag.* 16 567–583. 10.1080/09585190500051647

[B3] Ang S. Van Dyne L. Koh C. Ng K. Y. Templer K. J. Tay C. (2007). Cultural intelligence: its measurement and effects on cultural judgment and decision making, cultural adaptation and task performance. *Manage. Organ. Rev.* 3 335–371. 10.1111/j.1740-8784.2007.00082.x

[B4] Argyle M. Lu L. (1990). The happiness of extraverts. *Pers. Individ. Dif.* 11 1011–1017. 10.1016/0191-8869(90)90128-E

[B5] Arthur W. J. Bennett W. J. (1995). The International assignee: the relative importance of factors perceived to contribute to success. *Pers. Psychol.* 48 99–114. 10.1111/j.1744-6570.1995.tb01748.x

[B6] Asendorpf J. B. Wilpers S. (1998). Personality effects on social relationships. *J. Pers. Soc. Psychol.* 74 1531–1544. 10.1037/0022-3514.74.6.1531

[B7] Bandura A. (2002). Social cognitive theory in cultural context. *Appl. Psychol. Int. Rev.* 51 269–290. 10.1111/1464-0597.00092

[B8] Barrett L. F. Pietromonaco P. R. (1997). Accuracy of the five-factor model in predicting perceptions of daily social interactions. *Pers. Soc. Psychol. Bull.* 23 1173–1187. 10.1177/01461672972311005

[B9] Barrick M. R. Mount M. K. (1991). The Big Five Pers. dimensions and job performance: a meta-analysis. *Pers. Psychol.* 44 1–26. 10.1111/j.1744-6570.1991.tb00688.x

[B10] Bing M. N. Whanger J. C. Davison H. K. VanHook J. B. (2004). Incremental validity of the frame-of-reference effect in personality scale scores: a replication and extension. *J. Appl. Psychol.* 89 150–157. 10.1037/0021-9010.89.1.150 14769127

[B11] Black J. S. (1988). Work role transitions: a study of American expatriate managers in Japan. *J. Int. Bus. Stud.* 19 277–294. 10.1057/palgrave.jibs.8490383

[B12] Boyce C. J. Wood A. M. Daly M. Sedikides C. (2015). Personality change following unemployment. *J. Appl. Psychol.* 100 991–1011. 10.1037/a0038647 25664474

[B13] Brookfield Global Relocation Services (2012). *2012 Global Relocation Trends Survey.* Available at: http://knowledge.brookfieldgrs.com/content/insights_ideas-2012_GRTS">insights_ideas-2012_GRTS [accessed June 7 2012].

[B14] Caligiuri P. M. (2000). The Big Five personality characteristics as predictors of expatriate’s desire to terminate the assignment and supervisor-rated performance. *Pers. Psychol.* 53 67–88. 10.1111/j.1744-6570.2000.tb00194.x

[B15] Caspi A. Roberts B. W. (2001). Target article: personality development across the life course: the argument for change and continuity. *Psychol. Inq.* 12 49–66. 10.1207/S15327965PLI1202_01

[B16] Chan C. Schmitt N. (2000). Interindividual differences in intraindividual changes in proactivity during organizational entry: a latent growth modeling approach to understanding newcomer adaptation. *J. Appl. Psychol.* 85 190–210. 10.1037//0021-9010.85.2.190 10783536

[B17] Chan D. (2002). “Latent growth modeling,” in *Measuring and Analyzing Behavior in Organizations: Advances in Measurement and Data Analysis*, eds Drasgow F. Schmitt N. (San Francisco, CA: Jossey Bass), 302–349.

[B18] Chen G. Kirkman B. L. Kim K. Farh C. I. C. Tangirala S. (2010). When does cross-cultural motivation enhance expatriate effectiveness? A multilevel investigation of the moderating roles of subsidiary support and cultural distance. *Acad. Manage. J.* 53 1110–1130. 10.5465/AMJ.2010.54533217

[B19] Clausen J. A. Gilens M. (1990). Personality and labor force participation across the life course: a longitudinal study of women’s careers. *Sociol. Forum* 5 595–618. 10.1007/BF01115393

[B20] Costa P. T. Jr. McCrae R. R. (1992). *Revised NEO Personality Inventory (NEO PI-R) and NEO Five-Factor Inventory (NEO-FFI) Professional Manual.* Odessa, FL: Psychological Assessment Resources.

[B21] Credé M. Niehorster S. (2012). Adjustment to college as measured by the Student Adaptation to College Questionnaire: a quantitative review of its structure and relationships with correlates and consequences. *Educ. Psychol. Rev.* 24 133–165. 10.1007/s10648-011-9184-5

[B22] DeSimone J. A. Harms P. D. DeSimone A. J. (2015). Best practice recommendations for data screening. *J. Organ. Behav.* 36 171–181. 10.1002/job.1962

[B23] DeYoung C. G. Quilty L. C. Peterson J. B. (2007). Between facets and domains: 10 aspects of the Big Five. *J. Pers. Soc. Psychol.* 93 880–896. 10.1037/0022-3514.93.5.880 17983306

[B24] Donnellan M. B. Oswald F. L. Baird B. M. Lucas R. E. (2006). The mini-IPIP scales: tiny-yet-effective measures of the Big Five factors of personality. *Psychol. Assess.* 18 192–203. 10.1037/1040-3590.18.2.192 16768595

[B25] Eaton L. G. Funder D. C. (2003). The creation and consequences of the social world: an interactional analysis of extraversion. *Eur. J. Pers.* 17 375–395. 10.1002/per.477

[B26] Fraley R. C. Roberts B. W. (2005). Patterns of continuity: a dynamic model for conceptualizing the stability of individual differences in psychological constructs across the life course. *Psychol. Rev.* 112 60–74. 10.1037/0033-295X.112.1.60 15631588

[B27] Funder D. C. (2008). “Persons, situations, and person-situation interactions,” in *Handbook of Personality: Theory and Research*, eds John O. P. Robins R. W. Pervin L. A. (New York, NY: Guilford Press), 568–580.

[B28] Headey B. Wearing A. (1989). Personality, life events, and subjective well-being: toward a dynamic equilibrium model. *J. Pers. Soc. Psychol.* 57 731–739. 10.1037/0022-3514.57.4.731

[B29] Heller D. Ferris D. Brown D. Watson D. (2009). The influence of work personality on job satisfaction: incremental validity and mediation effects. *J. Pers.* 77 1051–1084. 10.1111/j.1467-6494.2009.00574.x 19558443

[B30] Hom P. W. Griffeth R. W. (1991). Structural equations modeling test of a turnover theory: cross-sectional and longitudinal analyses. *J. Appl. Psychol.* 76 350–366. 10.1037//0021-9010.76.3.350

[B31] Huang J. L. Bowling N. A. Liu M. Li Y. (2014a). Detecting insufficient effort responding with an infrequency scale: evaluating validity and participant reactions. *J. Bus. Psychol.* 30 299–311. 10.1007/s10869-014-9357-6

[B32] Huang J. L. Liu M. Bowling N. A. (2014b). Insufficient effort responding: examining an insidious confound in survey data. *J. Appl. Psychol.* 100 828–845. 10.1037/a0038510 25495093

[B33] Huang J. L. Ryan A. M. Zabel K. L. Palmer A. (2014c). Personality and adaptive performance at work: a meta-analytic investigation. *J. Appl. Psychol.* 99 162–179. 10.1037/a0034285 24016205

[B34] Huang J. L. Curran P. G. Keeney J. Poposki E. M. DeShon R. P. (2012). Detecting and deterring insufficient effort respond to surveys. *J. Bus. Psychol.* 27 99–114. 10.1007/s10869-011-9231-8

[B35] Huang J. L. Ford J. K. (2012). Driving locus of control and driving behavior: inducing change through driver training. *Transp. Res. F Traffic Psychol. Behav.* 15 358–368. 10.1016/j.trf.2011.09.002

[B36] Huang J. L. Ryan A. M. (2011). Beyond personality traits: a study of personality states and situational contingencies in customer service jobs. *Pers. Psychol.* 64 451–488. 10.1111/j.1744-6570.2011.01216.x

[B37] Hunthausen J. M. Truxillo D. M. Bauer T. N. Hammer L. B. (2003). A field study of frame-of-reference effects on personality test validity. *J. Appl. Psychol.* 88 545–551. 10.1037/0021-9010.88.3.545 12814302

[B38] Jundt D. K. Shoss M. Huang J. L. (2015). Individual adaptive performance in organizations: a review. *J. Organ. Behav.* 36 S53–S71. 10.1002/job.1955

[B39] Kaplan D. (2009). *Structural Equation Modeling: Foundations and Extensions.* Thousand Oaks, CA: Sage.

[B40] Kline R. B. (2005). *Principles and Practice Structural Equation Modeling.* New York, NY: Guilford Publications, Inc.

[B41] Kristof-Brown A. Guay R. P. (2011). “Person–environment fit,” in *APA Handbook of Industrial and Organizational Psychology: Maintaining, Expanding, and Contracting the Organization*, ed. Zedeck S. (Washington, DC: American Psychological Association), 3–50.

[B42] Lance C. E. Meade A. W. Williamson G. M. (2000). “We should measure change – and here’s how,” in *Physical Illness and Depression in Older Adults: Theory, Research, and Practice*, eds Williamson G. M. Schafer D. R. Pamelee P. A. (New York, NY: Plenum), 201–235.

[B43] Lehnart J. Neyer F. J. Eccles J. (2010). Long-term effects of social investment: the case of partnering in young adulthood. *J. Pers.* 78 639–670. 10.1111/j.1467-6494.2010.00629.x 20433633

[B44] Lewis M. (1999). “On the development of personality,” in *Handbook of Personality: Theory and Research*, eds Pervin L. A. John O. P. (New York, NY: Guilford Press), 327–346.

[B45] Lounsbury J. W. Saudargas R. A. Gibson L. W. Leong F. T. (2005). An investigation of broad and narrow Pers. traits in relation to general and domain-specific life satisfaction of college students. *Res. High. Educ.* 46 707–729. 10.1007/s11162-004-4140-6

[B46] Lucas R. E. Baird B. M. (2004). Extraversion and emotional reactivity. *J. Pers. Soc. Psychol.* 86 473–485. 10.1037/0022-3514.86.3.473 15008650

[B47] Lüdtke O. Roberts B. W. Trautwein U. Nagy G. (2011). A random walk down university avenue: life paths, life events, and personality trait change at the transition to university life. *J. Pers. Soc. Psychol.* 101 620–637. 10.1037/a0023743 21744977 PMC3596260

[B48] McCrae R. R. Terracciano A. (2005). Personality profiles of cultures: aggregate personality traits. *J. Pers. Soc. Psychol.* 89 407–425. 10.1037/0022-3514.89.3.407 16248722

[B49] Minbashian A. Wood R. E. Beckmann N. (2010). Task-contingent conscientiousness as a unit of personality at work. *J. Appl. Psychol.* 95 793–806. 10.1037/a0020016 20718521

[B50] Mischel W. (1968). *Personality and Assessment.* Hoboken, NJ: John Wiley and Sons.

[B51] Mischel W. (1973). Toward a cognitive social learning reconceptualization of personality. *Psychol. Rev.* 80 252–283. 10.1037/h0035002 4721473

[B52] Mroczek D. K. Spiro A. (2007). Personality change influences mortality in older men. *Psychol. Sci.* 18 371–376. 10.1111/j.1467-9280.2007.01907.x 17576273 PMC2643121

[B53] Naumann E. (1992). A conceptual model of expatriate turnover. *J. Int. Bus. Stud.* 23 499–531. 10.1057/palgrave.jibs.8490277

[B54] Neyer F. J. Lehnart J. (2007). Relationships matter in personality development: evidence from an 8-year longitudinal study across young adulthood. *J. Pers.* 75 535–568. 10.1111/j.1467-6494.2007.00448.x 17489891

[B55] Ones D. S. Viswesvaran C. (1999). Relative importance of personality dimensions for expatriate selection: a policy capturing study. *Hum. Perform.* 12 275–294. 10.1080/08959289909539872

[B56] Peltokorpi V. (2008). Cross-cultural adjustment of expatriates in Japan. *Int. J. Hum. Resour. Manag.* 19 1588–1606. 10.1080/09585190802294903

[B57] Roberts B. W. (1997). Plaster or plasticity: are adult work experiences associated with personality change in women? *J. Pers.* 65 205–232. 10.1111/j.1467-6494.1997.tb00953.x 9226940

[B58] Roberts B. W. Caspi A. Moffitt T. E. (2001). The kids are alright: growth and stability in personality development from adolescence to adulthood. *J. Pers. Soc. Psychol.* 81 670–683. 10.1037/0022-3514.81.4.670 11642353

[B59] Roberts B. W. Donahue E. M. (1994). One personality, multiple selves: integrating personality and social roles. *J. Pers.* 62 199–218. 10.1111/j.1467-6494.1994.tb00291.x 8046573

[B60] Roberts B. W. Mroczek D. (2008). Personality trait change in adulthood. *Curr. Dir. Psychol. Sci.* 17 31–35. 10.1111/j.1467-8721.2008.00543.x 19756219 PMC2743415

[B61] Roberts B. W. Wood D. (2006). “Personality development in the context of the neo-socioanalytic model of personality,” in *Handbook of Personality Development*, eds Mroczek D. K. Little T. D. (Mahwah, NJ: Lawrence Erlbaum Associates Publishers), 11–39.

[B62] Roberts B. W. Wood D. Caspi A. (2008). “The development of personality traits in adulthood,” in *Handbook of Personality Psychology: Theory and Research*, eds John O. P. Robins R. W. Pervin L. A. (New York, NY: Guilford Press), 375–398.

[B63] Ross S. E. Neibling B. C. Heckert T. M. (1999). Sources of stress among college students. *Coll. Stud. J.* 33 312–317.

[B64] Schmit M. J. Ryan A. M. Stierwalt S. L. Powell A. B. (1995). Frame-of-reference effects on personality scale scores and criterion-related validity. *J. Appl. Psychol.* 80 607–620. 10.1037/0021-9010.80.5.607

[B65] Schneider B. (1987). The people make the place. *Pers. Psychol.* 40 437–453. 10.1111/j.1744-6570.1987.tb00609.x

[B66] Shaffer J. A. Postlethwaite B. E. (2012). A matter of context: a meta-analytic investigation of the relative validity of contextualized and noncontextualized personality measures. *Pers. Psychol.* 65 445–493. 10.1111/j.1744-6570.2012.01250.x

[B67] Shaffer M. A. Harrison D. A. Gilley K. M. (1999). Dimensions, determinants, and differences in the expatriate adjustment process. *J. Int. Bus. Stud.* 30 557–581. 10.1057/palgrave.jibs.8490083

[B68] Shaffer M. A. Harrison D. A. Gregersen H. Black J. S. Ferzandi L. A. (2006). You can take it with you: individual differences and expatriate effectiveness. *J. Appl. Psychol.* 91 109–125. 10.1037/0021-9010.91.1.109 16435942

[B69] Siegler I. C. Costa P. T. Brummett B. H. Helms M. J. Barefoot J. C. Williams R. B. (2003). Patterns of change in hostility from college to midlife in the UNC Alumni Heart Study predict high-risk status. *Psychosom. Med.* 65 738–745. 10.1097/01.PSY.0000088583.25140.9C 14508014

[B70] Snyder M. (1974). Self-monitoring of expressive behavior. *J. Pers. Soc. Psychol.* 30 526–537. 10.1037/h0037039

[B71] Specht J. Egloff B. Schmukle S. C. (2011). Stability and change of personality across the life course: the impact of age and major life events on mean-level and rank-order stability of the Big Five. *J. Pers. Soc. Psychol.* 101 862–882. 10.1037/a0024950 21859226

[B72] Sri Ramalu S. Rose R. C. Uli J. Kumar N. (2010). Personality and cross-cultural adjustment among expatriate assignees in Malaysia. *Int. Bus. Res.* 3 96–104. 10.5539/ibr.v3n4p96

[B73] Swagler M. A. Jome L. M. (2005). The effects of personality and acculturation on the adjustment of North American sojourners in Taiwan. *J. Couns. Psychol.* 52 527–536. 10.1037/0022-0167.52.4.527

[B74] Templer K. J. Tay C. Chandrasekar N. A. (2006). Motivational cultural intelligence: realistic job preview, realistic living conditions preview, and cross-cultural adjustment. *Group Organ. Manage.* 31 154–173. 10.1177/1059601105275293

[B75] USNews (2012). *National Universities with Most International Students.* Available at: http://colleges.usnews.rankingsandRev.s.com/best-colleges/rankings/national-universities/most-international?src=stats">universities/most-Int?src=stats [accessed July 15 2012].

[B76] Vaidya J. G. Gray E. K. Haig J. Watson D. (2002). On the temporal stability of personality: evidence for differential stability and the role of life experiences. *J. Pers. Soc. Psychol.* 83 1469–1484. 10.1037/0022-3514.83.6.1469 12500825

[B77] Ward C. Okura Y. Kennedy A. Kojima T. (1998). The U-curve on trial: a longitudinal study of psychological and sociocultural adjustment during cross-cultural transition. *Int. J. Intercult. Relat.* 22 277–291. 10.1016/S0147-1767(98)00008-X

[B78] White J. K. Hendrick S. S. Hendrick C. (2004). Big five personality variables and relationship constructs. *Pers. Individ. Dif.* 37 1519–1530. 10.1016/j.paid.2004.02.019

[B79] Wright J. C. Mischel W. (1987). A conditional approach to dispositional constructs: the local predictability of social behavior. *J. Pers. Soc. Psychol.* 53 1159–1177. 10.1037/0022-3514.53.6.1159 3694455

[B80] Ying Y. (2005). Variation in acculturative stressors over time: a study of Taiwanese students in the United States. *Int. J. Intercult. Relat.* 29 59–71. 10.1016/j.ijintrel.2005.04.003

